# Temporally resolved growth patterns reveal novel information about the polygenic nature of complex quantitative traits

**DOI:** 10.1111/tpj.17092

**Published:** 2024-10-27

**Authors:** Dorothy D. Sweet, Sara B. Tirado, Julian Cooper, Nathan M. Springer, Cory D. Hirsch, Candice N. Hirsch

**Affiliations:** ^1^ Department of Agronomy and Plant Genetics University of Minnesota Saint Paul Minnesota 55108 USA; ^2^ Department of Plant Pathology University of Minnesota Saint Paul Minnesota 55108 USA; ^3^ Department of Plant and Microbial Biology University of Minnesota Saint Paul Minnesota 55108 USA

**Keywords:** canopy cover, Fréchet distance, genome‐wide association studies, genotype‐by‐environment interaction, plant height, unoccupied aerial vehicles, *Zea mays* L

## Abstract

Plant height can be an indicator of plant health across environments and used to identify superior genotypes. Typically plant height is measured at a single timepoint when plants reach terminal height. Evaluating plant height using unoccupied aerial vehicles allows for measurements throughout the growing season, facilitating a better understanding of plant‐environment interactions and the genetic basis of this complex trait. To assess variation throughout development, plant height data was collected from planting until terminal height at anthesis (14 flights 2018, 27 in 2019, 12 in 2020, and 11 in 2021) for a panel of ~500 diverse maize inbred lines. The percent variance explained in plant height throughout the season was significantly explained by genotype (9–48%), year (4–52%), and genotype‐by‐year interactions (14–36%) to varying extents throughout development. Genome‐wide association studies revealed 717 significant single nucleotide polymorphisms associated with plant height and growth rate at different parts of the growing season specific to certain phases of vegetative growth. When plant height growth curves were compared to growth curves estimated from canopy cover, greater Fréchet distance stability was observed in plant height growth curves than for canopy cover. This indicated canopy cover may be more useful for understanding environmental modulation of overall plant growth and plant height better for understanding genotypic modulation of overall plant growth. This study demonstrated that substantial information can be gained from high temporal resolution data to understand how plants differentially interact with the environment and can enhance our understanding of the genetic basis of complex polygenic traits.

## Significance Statement

Unmanned aerial vehicles were used to collect high temporal resolution morphological data of maize plants and model growth curves of genetically diverse inbred lines. These growth curves provide a more complete understanding of how plants differentially interact with the environment. Dissecting the genetic basis of growth curves also provides a deeper understanding of the genetic basis of complex polygenic traits.

## INTRODUCTION

Advancements in crop improvement and production are essential to keep up with the current rates of population growth and changing climate. Over the past few decades, progress in sequencing technology has driven the development and implementation of genomic sequencing and marker platforms that can speed up crop improvement (Yang et al., 2020). Advances in the collection of accurate, high‐resolution phenotypic traits across many crop varieties and environments are also essential to meet this demand (Herr et al., 2023). Most current methods of trait acquisition are time‐consuming, laborious, and only collected at a single timepoint at the end of the season, which leaves high‐throughput phenotyping in field environments as a major bottleneck in crop improvement (Silva‐Perez et al., 2018; White et al., 2012).

Plant height is an important trait in maize (*Zea mays* L.) breeding programs and can be used to monitor growth rates (Wang et al., 2018), assess plant health (Dhami, 2019), and predict yield (Boomsma et al., 2010; Yin, McClure, et al., 2011). Measurements of plant height have also been used to determine field spatial variability when evaluating abiotic influences such as nitrogen content (Katsvairo et al., 2003; Yin, Jaja, et al., 2011). These findings suggested plant height and estimated growth rates can be used as metrics to identify agronomically superior cultivars in plant breeding programs and to develop management practices to account for spatial variation in production fields. Unoccupied aerial vehicles (UAVs) are particularly useful for collecting maize plant height information since manual collection using rulers is not only time‐consuming and often only collected at the end of the growing season, but is also associated with error due to viewing angles of data collectors or random sampling error (Tirado et al., 2020). UAVs can also collect data when issues such as wet soil and plant lodging prevent ground vehicles from navigating plots (Tirado et al., 2021).

Detection of plant height from UAV imagery has advanced through the use of structure from motion (SfM), which can create 3D reconstructions from overlapping overhead images (James & Robson, 2012). The 3D reconstructions developed through SfM can be used to create orthomosaics and digital elevation models, which can be used to extract traits such as plant height. Multiple examples of accurate plant height estimates using UAV imagery have been shown in crops such as barley (*Hordeum vulgare* L.) (Bendig et al., 2014; Herzig et al., 2021), cabbage (*Brassica oleracea* var. *capitata* L.) (Moeckel et al., 2018), cotton (*Gossypium hirsutum* L.) (Feng et al., 2019; Liu et al., 2020), eggplant (*Solanum melongena* L.) (Moeckel et al., 2018), faba bean (*Vicia faba* L.) (Ji et al., 2022), maize (Adak, Murray, Božinović, et al., 2021; Anderson et al., 2019; Anthony et al., 2014; Geipel et al., 2014; Grenzdörffer, 2014; Letsoin et al., 2023; Malambo et al., 2018; Shi et al., 2016; Su et al., 2019; Tirado et al., 2020; Varela et al., 2017), potato (*Solanum tuberosum* L.) (de Jesus Colwell et al., 2021; Njane et al., 2023; Xie et al., 2022), rapeseed (*Brassica napus* L.) (Xie et al., 2021), sorghum (*Sorghum bicolor* L.) (Chang et al., 2017; Gano et al., 2021; Shi et al., 2016; Watanabe et al., 2017), soybean (*Glycine max* L.) (Li et al., 2022), tomato (*Solanum lycopersicum* L.) (Moeckel et al., 2018), and wheat (*Triticum aestivum* L.) (Holman et al., 2016; Madec et al., 2017; Michalski et al., 2018; Volpato et al., 2021). Studies on plant height using UAVs have shown variable levels of success when compared to manual measurements due to plant structure, field layout, and improvements in best practices as more research was completed (Holman et al., 2016; Sweet et al., 2022).

A major advantage to using UAVs to measure traits such as plant height is the ability to collect trait data at regular intervals throughout the growing season. High temporal resolution data collection facilitates a better understanding of end‐of‐season traits that are the culmination of a growing season's worth of genotype‐by‐environment interactions (de Jesus Colwell et al., 2021) and genotype‐by‐flight interactions (Adak et al., 2024). Such temporal measurements have been completed on canopy cover and biomass in soybeans (Freitas Moreira et al., 2021; Herrero‐Huerta & Rainey, 2019; Li et al., 2022; Moreira et al., 2019); plant height, canopy cover, growth dynamics, and yield prediction in barley (Herzig et al., 2021); plant height and canopy cover in banana (*Musa paradisiaca* L.) (Aeberli et al., 2023); nutrient status, canopy cover, canopy volume, and plant height in potatoes (de Jesus Colwell et al., 2021; Liu et al., 2021); plant height in cotton (Andrea et al., 2023); and plant height in eggplant, tomato, cabbage (Moeckel et al., 2018). In maize, multi‐temporal measurements of multiple traits have been used to explain the effect of phosphorus on plant growth and final yield (Herrmann et al., 2020; Pedersen et al., 2021), phenotypic variation due to genotypic background differences (Adak et al., 2024; Adak, Anderson, & Murray, 2023; Adak, Murray, & Anderson, 2023; Adak, Murray, Božinović, et al., 2021; Han et al., 2019; Pugh et al., 2018), phenotypic variation due to environmental factors such as drought (Machado et al., 2002) or high‐speed wind events (Tirado et al., 2021), and canopy cover (Jin et al., 2020; Lu et al., 2021).

In addition to capturing phenotypic responses to the environment, high temporal resolution phenotyping can facilitate a more complete understanding of the genetic factors controlling a trait. Previous research looking at the genetic factors controlling maize plant height was completed with single measurements of terminal plant height due to the labor intensity of manual height collection (Peiffer et al., 2014). A study evaluating terminal plant height of many maize inbred lines in multiple environments found plant height was highly heritable and predictable with models, but also highly polygenic with many small effect alleles contributing to the overall height, which are difficult to identify (Peiffer et al., 2014). Subsequent studies with higher temporal data collection were able to identify quantitative trait locus (QTLs) associated with early and mid‐season plant height, growth rates, and growth curves (Adak, Anderson, & Murray, 2023; Adak, Murray, & Anderson, 2023; Adak, Conrad, Chen, et al., 2021; Wang et al., 2019). These studies varied in the number of time points at which plant height was measured, the genetic diversity of the material used, and the number of environments evaluated. For example, Adak, Anderson, and Murray (2023) and Adak, Murray, and Anderson (2023) collected RGB images of 280 maize hybrids over 15 time points, and identified 241 genome‐wide association study (GWAS) peaks over 36 temporal phenotypes. These early studies provided valuable information in understanding genetic variation in growth rates, yet understanding of the impact of genotype, environment, and genotype‐by‐environment interaction (G × E) on temporally resolved growth curves is still lacking due to the limited number of growth environments and/or genotypes included in the studies.

Here we report on high temporal resolution phenotyping of a panel of over 500 diverse maize lines from the Wisconsin diversity panel (Hansey et al., 2011) that represent the breadth of variation in temperate maize germplasm grown over four growing environments between 2018 and 2021. The objectives of this study were (i) to determine how much plant height variance is explained by different experimental factors (i.e., genotype, environment, and G × E) throughout development, (ii) to identify patterns of growth rates within and between growing seasons and assess the consistency of these growth patterns, (iii) to map the genetic basis of plant height and growth rate throughout vegetative growth across multiple environments, and (iv) to evaluate the differences and relative utility of using different traits (i.e., plant height versus canopy cover) to generate plant growth curves.

## RESULTS AND DISCUSSION

### LOESS curves fit to the data break the growing season into three phases

**Figure 1:**
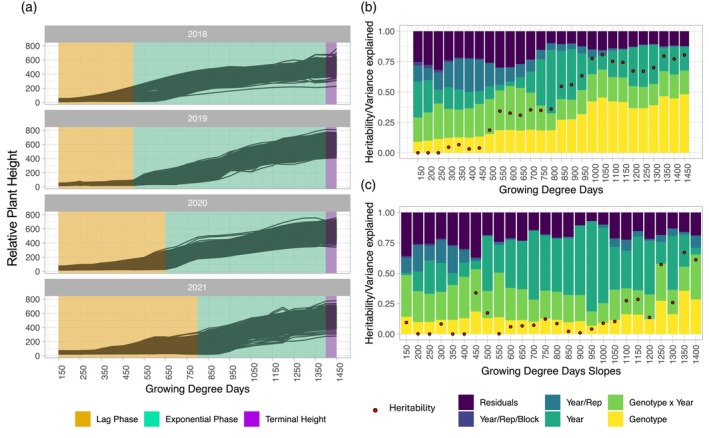
Temporal plant height and growth rate analysis of variance. (a) LOESS curves of plant height are broken into three phases of the growing season based on the performance of genotypes in each year. (b) Variance explained and heritability at each plant height timepoint throughout the growing season. (c) Variance explained and heritability at each growth rate timepoint throughout the growing season.

Flights were conducted at 14 timepoints in 2018, 27 timepoints in 2019, 12 timepoints in 2020, and 11 timepoints in 2021 to obtain plant height data throughout each growing season (Tables S1 and S2). Plant height values were normalized across flights in order to obtain relative plant height values across flights and environments, but plant height values were not normalized to any real‐world units. In order to directly and quantitatively compare growth curves among genotypes and years, a method of standardization was needed to align similar timepoints from 1 year to the next. Converting the dates of flights to growing degree days (GDDs) made it possible to compare growth stages across years while accounting for different planting dates and temperatures across the years (Table S3). LOESS curves were fit to the data from each plot, and plant height in 50 GDD increments throughout the growing season was predicted from the curves (Figure S1; Table S4). This method has been used in previous studies to allow for direct comparisons regardless of the actual flight dates across the years (Cooper et al., 2024; Sweet et al., 2022; Tirado et al., 2021). The growth curves were annotated to denote lag phase, exponential growth phase, and terminal height (Figure 1a). These phases were determined based on the slope of the growth curve between timepoints of plant height. The transition from the lag phase to exponential growth occurred when the average slope of the plots passed 0.3. Terminal height occurred when the average slope of the plots decreased back under 0.1. This dissection of the growing season is important as both plant growth and grain yield are differentially affected by temperature and precipitation throughout the growing season, especially when the variation occurs early in the season (Butts‐Wilmsmeyer et al., 2019; Claassen & Shaw, 1970; Dodig et al., 2021).

The beginning of the exponential growth phase differed between years, with 2018 and 2019 reaching the phase as early as 500 GDDs and 2021 reaching exponential growth as late as 800 GDDs (Figure 1a). This is likely due to differences in the speed of GDD accumulation, early season precipitation, and planting time. The planting dates in 2018 and 2019 were later (05/14/2018 and 05/30/2019), while 2020 and 2021 were planted earlier in the calendar year (05/07/2020 and 05/06/2021) allowing for a more gradual accumulation of GDDs (Figure S1b; Table S5). The most precipitation prior to the exponential growth period was accumulated in 2020 with 15.7 cm while the rest of the years had accumulated only 7.7, 8.0, and 9.0 cm in 2018, 2019, and 2021, respectively (Table S5). Additionally, 2021 had a long dry spell (22 days with minimal to no rain) from 219 to 724 GDDs while other years showed no significant water stress during that time.

### Heritability of plant height increased over time, while growth rate was largely explained by year for much of the growing season

Highly variable plant height and growth rate values across years indicated that aspects of the environment had a large phenotypic impact in addition to the genetic background of the material. An analysis of variance (anova) showed that the percent variance explained by genotype gradually increased throughout the growing season, which coincided with increased heritability throughout the season (Figure 1b). This trend is similar to previously observed patterns using temporal vegetation indices (Adak et al., 2024) and temporal plant height in maize (Pugh et al., 2018). The accuracy of genomic prediction using genome‐wide single nucleotide polymorphism (SNP) markers also increased over time (Figure S2), consistent with an increase in variance explained by genotype.

In contrast to genotype, replicate explained the greatest proportion of the observed variation early in the growing season starting at 200 GDD due to limited overall variation early in the season as well as variable soil temperature and moisture conditions early in the growing season resulting in more spatial variation within the field (Figure 1b). As the growing seasons continued there was almost no variation explained by replicate, and by 1150 GDDs the percent variation explained (PVE) for replicate did not exceed 0.8% for the remainder of the growing season. Pugh et al. (2018) also showed large variation due to replicate earlier in the season for maize planted at the optimal time that decreased as the season continued. Genotype‐by‐year interaction also explained more variance early in the growing season when the difference in days to accumulate early GDDs was highly variable (Figure S1b).

Throughout the exponential growth phase, a larger portion of the variance is partitioned to the year term than any other time in the growing season. During this phase of vegetative growth, stalk elongation can be severely affected by environmental factors such as water availability (Claassen & Shaw, 1970). Indeed, the amount of water accumulation through this phase of the growing season differed nearly three fold over the four seasons this experiment was grown, ranging from 6.3 cm in 2021 to 17.2 cm in 2018 (Minnesota Department of Natural Resources, 2024) (Table S5). The timing of precipitation events also varied between the years contributing to differences in the growth patterns across the years (Figure S3).

Rate of growth, calculated as the slope between plant heights, generally had less distinctive patterns than plant height in how variance was partitioned (Figure 1c). Overall, a much larger proportion of the variation was explained by year and genotype‐by‐year interaction, demonstrating that growth rate was less dependent on the genotype and influenced more by the environment. This was particularly evident during the exponential growth phase when 17–74% of the total variation was explained by year. Similar to plant height, the highest PVE by replicate for growth rate also occurred early in the season when soil temperature and other soil conditions were more variable.

### Clustering revealed three growth patterns that fluctuated within genotype from year to year

**Figure 2:**
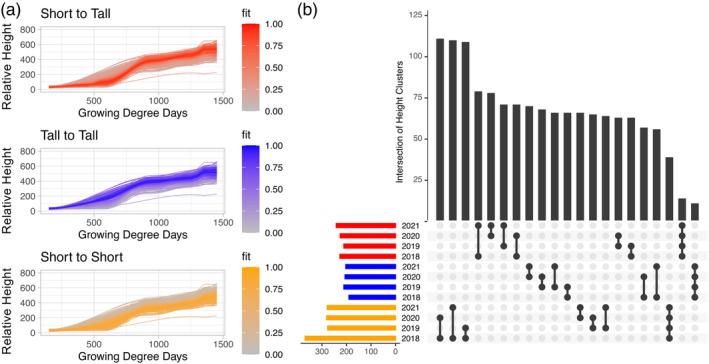
Fuzzy c‐means clustering of LOESS growth curves. (a) Fuzzy c‐means clusters of 2018 LOESS growth curves with shading equating goodness of fit for each curve into the specified cluster. (b) Upset plot showing overlap of genotypes between growth curve clusters across years. Each curve is classified into a cluster based on the highest fit for each curve. Cluster types are represented by identifying colors (yellow: tall to tall; blue: short to short; red: short to tall).

The anova partitioned variation at each individual timepoint. To further understand the significant variance explained by year and genotype‐by‐year interaction on the full growth curve, the growth curves were clustered within each year using fuzzy c‐means clustering, and patterns were compared within and among years. Fuzzy c‐means clustering places each curve into each cluster with various degrees of fit into the clusters ranging from 0 to 1 (Table S6). This approach allowed curves to be grouped into defined clusters based on goodness of fit, while also providing information on how similar the genotypes within a group are to each other (Han et al., 2019). The number of clusters was chosen based on the distribution of the largest goodness of fit value for each curve (Figure S4) with 3 clusters having a fairly normal distribution. The three clusters were characterized as genotypes that start tall relative to other genotypes in the experiment and end tall, genotypes that start short and end short, and genotypes that start short and end tall (Figure 2a), with some years fitting these patterns better than others (Figure S5). In order to compare groups across years, each curve was assigned to a group based on its largest goodness of fit value. A comparison of distinct curves across all 4 years showed that genotypes do not always have the same growth pattern (Figure 2b; Table S7). The short‐to‐short pattern kept the most consistent genotypes with 39 that are present in all 4 years out of the 403 genotypes with that pattern in at least 1 year. Despite the low percentage of genotypes with the same pattern across all 4 years, the high recurrence of genotypes within single pairings showed that some years are more similar than others. For instance, between 2018 and 2020, 111 genotypes stayed in the short to short growth pattern out of the 158 genotypes in that pattern in 2018, meaning 70% of the genotypes with a short to short pattern in 2018 had a short to short pattern in 2020 (Figure 2b).

### Fréchet distances of growth curves provided more information on plasticity and stability than terminal height

**Figure 3:**
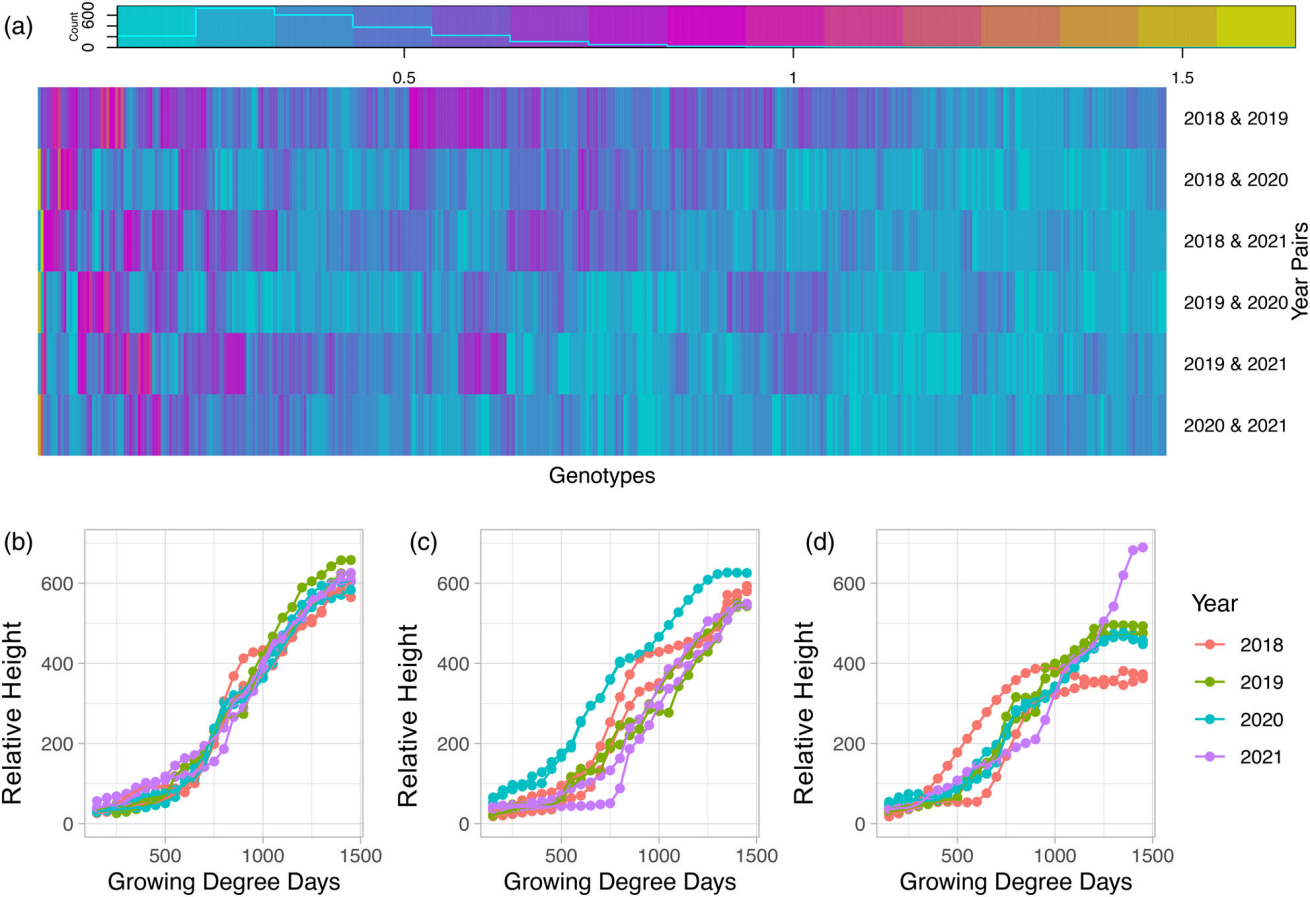
Fréchet distances for each genotype present in all 4 years. (a) Pairwise Fréchet distance values comparing plant height growth curves of the same genotype across different years. (b) Example genotype (PHG47) representing genotypes with consistently low Fréchet distance values and low variation in terminal height. (c) Example genotype (YING‐55) representing genotypes with high Fréchet distance values and low variation in terminal height. (d) Example genotype (PHN66) representing genotypes with high Fréchet distance values and high variation in terminal height.

**Figure 4:**
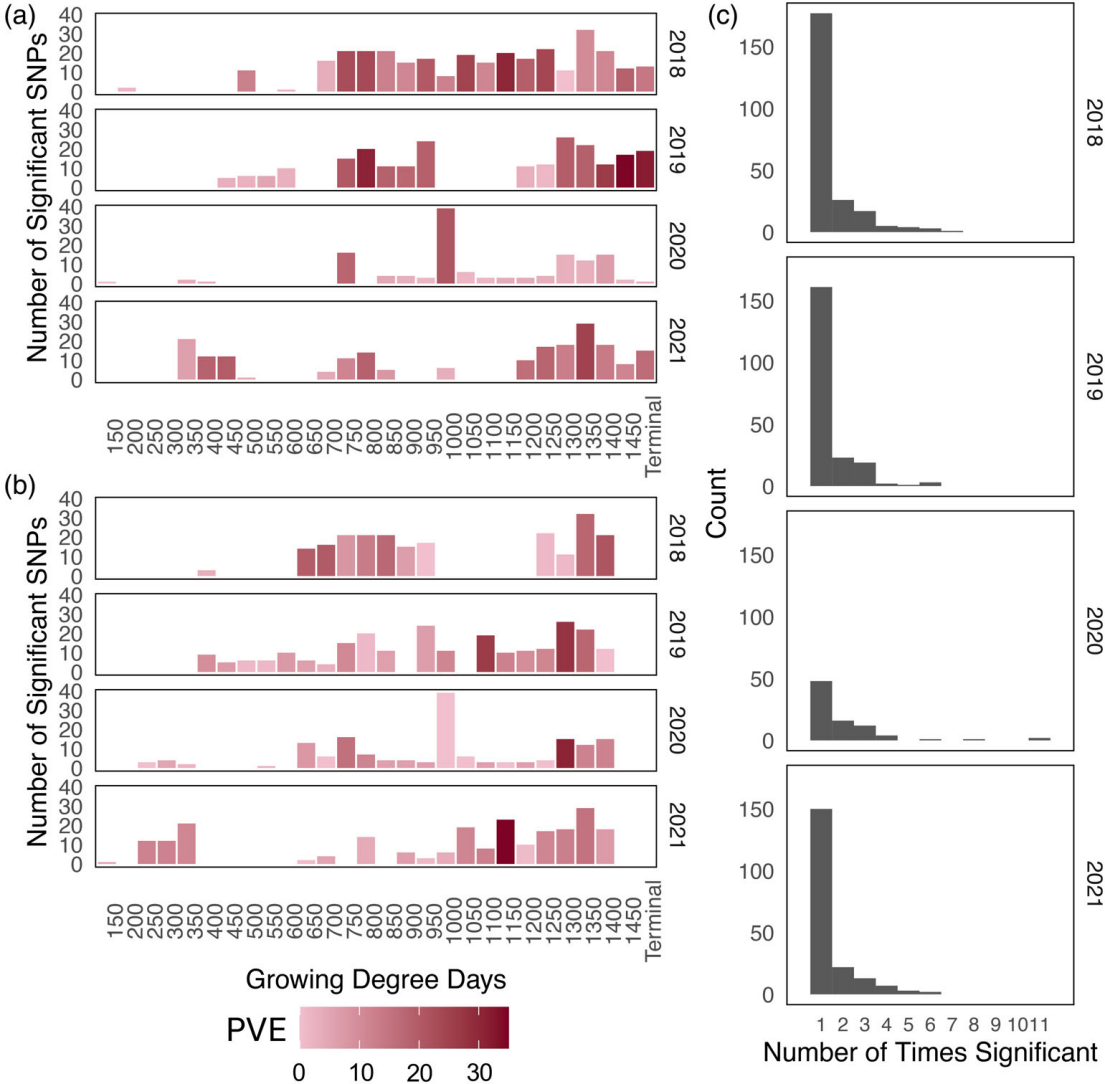
Significant single nucleotide polymorphisms (SNPs) identified with genome‐wide association study (GWAS) across development. (a) Results from plant height GWAS at each growing degree day interval throughout the growing season shaded by percent variance explained (PVE) of the significant SNPs at that timepoint. (b) Results from growth rate GWAS at each growing degree day interval throughout the growing season shaded by PVE of the significant SNPs at that interval. (c) Number of timepoints (height) or intervals (rate) in which a SNP was identified as significant within a year.

Clustering the genotypes into groups gave an idea of how similar the growth curves were between different genotypes, but we were also interested in a quantitative measurement of the similarity of the curve for each genotype across years. Other studies have employed practices such as “phenotypic similarity trees” to calculate similarity distances based on many independent phenotypic traits (Chen et al., 2014). As our data is a continuous series of data points that make up a growth curve we instead evaluated the curve similarity using the Fréchet distance. The Fréchet distance is a measurement of similarity that takes location and order of points along the curve into account and is often described as the shortest leash possible when a person walks on one curve and their dog walks on the other (Fréchet, 1906). In this case, it can be used to observe general trends between years, as well as the plasticity of specific genotypes across the years. To assess overall differences among years, each year was compared to every other year with average Fréchet distances over all genotypes varying remarkably (Figure 3a; Figure S6; Table S8). Some of the genotypes resulted in consistently low Fréchet distances between year pairings (e.g., Figure 4b), while other genotypes (e.g., Figure 4c,d) demonstrated high variability across years with some year pairings performing more similarly and others performing very differently. Eighteen environmental parameters in daily increments throughout the growing season were used to calculate pairwise environmental correlations between each combination of years (Table S5). A strong relationship between the pair‐wise average Fréchet distances and the pair‐wise environmental correlations was observed (Pearson correlation coefficient = 0.80; Figure S6). The very large average Fréchet distance between 2018 and 2019 was likely due to these years having the largest difference in GDD accumulation across all 4 years. In 2018, GDDs began to accumulate earlier, and by mid to late August there was a large difference in the number of GDDs accumulated (August 17, 2018–2248.5 GDDs, August 21, 2019–1650.5 GDDs) (Figure S1b). Conversely, in 2020 and 2021, which has the smallest average Fréchet distance across year pairs (0.3650), there is a very similar accumulation of GDDs relative to calendar day in the growing season (July 22, 2020–1340 GDDs, July 23, 2021–1421 GDDs). This indicates that while GDDs are a good indicator of developmental stages, they are not necessarily linked to plant growth.

Fréchet distances were also used to assess differences in overall growth curve topology within genotypes across year pairs as a metric of growth stability for individual genotypes. A wide range of Fréchet distances were observed across all of the genotypes (average 0.209–1.055 across all year pairs, Table S8), reflecting variable environmental responsiveness of the different genotypes in this population throughout vegetative development. To determine the impact of variable growth patterns across years on terminal height, the Fréchet distances across years were compared to variance in terminal plant height. The average variance of terminal plant height across years ranged from 4 to 13 035, also reflective of the variability in environmental responsiveness across the genotypes. While a wide range of variance in both growth curve and terminal height stability was observed, there was not a strong relationship between average stability across environments for growth curve and terminal plant height (Pearson correlation coefficient = 0.36). Thus the relative variance in end‐of‐season traits does not always reflect the variance in the growth and development required to reach the end‐of‐season trait. Some genotypes were stable for both growth curves (low Fréchet distance) and variance in terminal plant height (e.g., Figure 3b). Of the 100 most stable genotypes for terminal plant height (20th percentile), 26 of these were also in the 20th percentile for growth curve stability. In contrast, other genotypes had very stable terminal height, but their growth curves varied substantially between growing seasons (e.g., Figure 3c). Of the same 100 most stable genotypes for terminal plant height, 10 of these were in the top 80th percentile for Fréchet distance and did not reach the consistent terminal height in the same way each year. Most (*n* = 39) of the remaining genotypes with highly unstable growth curves (top 80th percentile) resulted in high variability in terminal height (top 80th percentile) (e.g., Figure 3d), and would likely be identified as having high plasticity across these growth environments regardless of analysis with terminal plant height or full growth curves. This analysis demonstrates that full growth curves give different, if not more complete, information about genotypic plasticity than terminal height, which has often been used (Mu et al., 2022).

### GWAS identified 669 significant SNPs throughout the growing season that were not identified at terminal height

To identify the genetic basis of the observed variation in growth curves across the genotypes in this study, GWAS were conducted on plant height and growth rate at 50 GDD windows throughout the growing season. Other studies have completed temporal GWAS with plant height in maize (Adak, Conrad, Chen, et al., 2021; Farfan et al., 2015; Wang et al., 2023) as well as vegetative indices (Adak, Kang, et al., 2023; Adak, Murray, Anderson, et al., 2021; Rodene et al., 2022; Wang et al., 2021); however, this study is unique by using a large diverse inbred panel in field settings over multiple years. This experimental design allowed a deeper understanding of how the genetics of complex traits such as plant height interact with the environment. GWAS completed for each plant height GDD, growth rate, and extracted terminal plant height revealed a total of 717 non‐redundant unlinked SNPs (Table S9). Of these SNPs, 48 were identified at terminal height (Figure 4a), with the majority of these 48 (*n* = 42) not identified at any other timepoint. Thus, the vast majority of the significant SNPs identified in this study (*n* = 669), would be missed if the GWAS was conducted only at the terminal time‐point as has been previously done (Wallace et al., 2016; Weng et al., 2011; Zhang et al., 2019). SNPs that were not identified in terminal height, but were identified earlier in the season may be helpful in improving early‐season plant resilience to detrimental weather effects such as low temperatures or high rainfall and may allow plants to outgrow competing weeds. Significant SNPs identified in early growth rates (Figure 4b) may be especially important as these SNPs appear to be particularly affected by the environment.

Plant height has long been documented as a complex, quantitative trait, even in studies that only assessed terminal variation (Wallace et al., 2016; Weng et al., 2011; Zhang et al., 2019). The large number (*n* = 717) of significant SNPs throughout the growing season in this study demonstrated further complexity in this trait than previously recognized with other studies identifying between 4 and 204 loci associated with plant height (Adak, Murray, Anderson, et al., 2021; Mazaheri et al., 2019; Peiffer et al., 2014; Wang et al., 2019; Weng et al., 2011; Zhang et al., 2019). Of the 717 significant SNPs identified in this study, 22 overlapped with previously identified plant height QTL (Table S10) (Adak, Murray, Anderson, et al., 2021; Mazaheri et al., 2019; Wang et al., 2019; Zhang et al., 2019). These overlapping QTL likely represent highly stable QTL with minimal QTL‐by‐background or QTL‐by‐environment effects. The limited number of loci in previous studies that did not overlap the loci identified in the current study could be false positives in those studies, loci that are not segregating in the germplasm used in the current study and so unable to be detected, or have a strong QTL‐by‐background or QTL‐by‐environment interaction. Conversely, there were substantially more novel loci identified in our study compared to these previous studies that were only able to be identified due to the use of high‐resolution temporal data. These novel significant loci had a range of biological functions related to cell division, cell elongation, general growth and development, stress, and environmental response, among other functions (Table S10). The cellular component gene ontology terms of chloroplast outer membrane and plastid outer membrane were significantly enriched in loci that were associated with plant height in the exponential growth phase, while binding functional terms were enrichment in loci associated with growth rate during the exponential growth phase (Table S11).

To assess how much of the total variation was captured by the SNPs that were identified as significant, the total PVE by the subset of significant SNPs at each time‐point was calculated. The PVE at any given timepoint‐by‐year combination ranged from 1.5 to 34.6% for height (Figure 4a) and from 0.0 to 27.8% for growth rate when looking at SNPs significant only at that individual timepoint‐by‐year combination (Figure 4b). PVE by significant SNPs at a single timepoint tended to explain more variance mid to late season in plant height (Figure 4a), which is consistent with the observed increase in heritability throughout the growing season (Figure 1b). We were also interested in the PVE at each timepoint using all significant SNPs across development to determine if these additional markers were able to explain substantially more variation. Indeed, PVE at any given timepoint‐by‐year combination calculated using all significant SNPs identified across time was much larger with PVE for height ranging from 2.0 to 70% and from 0.6 to 60.6% for growth rate. This demonstrates that high temporal resolution phenotyping facilitates a deeper understanding of the complete genetic basis of this complex trait. Furthermore, when looking at the variation in terminal plant height, all of the significant SNPs were able to explain up to 65% of the variation (51–65% across years). In contrast, only 17 to 36% of the variation in terminal plant height within a year was explained when using all SNPs identified as significant at terminal height in any of the years. To determine if this difference in percent variance explained was more than expected by chance, 669 random unlinked SNPs were added to the terminal plant height identified SNPs to total the same as the total number of SNPs in all timepoints and years. Using these SNPs, only 24 to 51% of the variation across the years was explained, indicating that the SNPs found significant throughout the season provided novel biologically relevant information about the end‐of‐season trait.

We were interested to know how many of the identified SNPs were closely tied to environmental interactions and if SNPs were impacting plant height for long periods of time. In order to ascertain this information, we looked into the number of times SNPs were found to be significant within years and if the same SNPs were significant across years. Of the total 717 significant SNPs, 175 were found to be significant at multiple timepoints, leaving most of the significant SNPs to only appear once (Figure 4c; Table S12). The 175 SNPs appeared from 2 to 11 separate times with most reappearances occurring within the same year, including 2 SNPs in 2020 that were significant at 11 different timepoints throughout development. Seven of these SNPs found at multiple timepoints in a single year overlapped with previously identified regions (Adak, Murray, Anderson, et al., 2021; Mazaheri et al., 2019; Wang et al., 2019; Zhang et al., 2019), further demonstrating the stability of these loci not only across backgrounds and environments but also throughout development. Six of the recurring SNPs were found to be significant over multiple years, five between 2018 and 2019 and one between 2019 and 2021. These SNPs may be less dependent on environmental factors and more generally impactful on growth. Two of these SNPs were found to be significant in multiple years toward the middle of the season during the exponential growth phase and are likely linked to stem elongation while four of them are significant at the end of the season and would be more likely to be tied to flowering time and picked up by studies only examining terminal height. Indeed, three of these four SNPs were also associated with terminal plant height.

**Figure 5:**
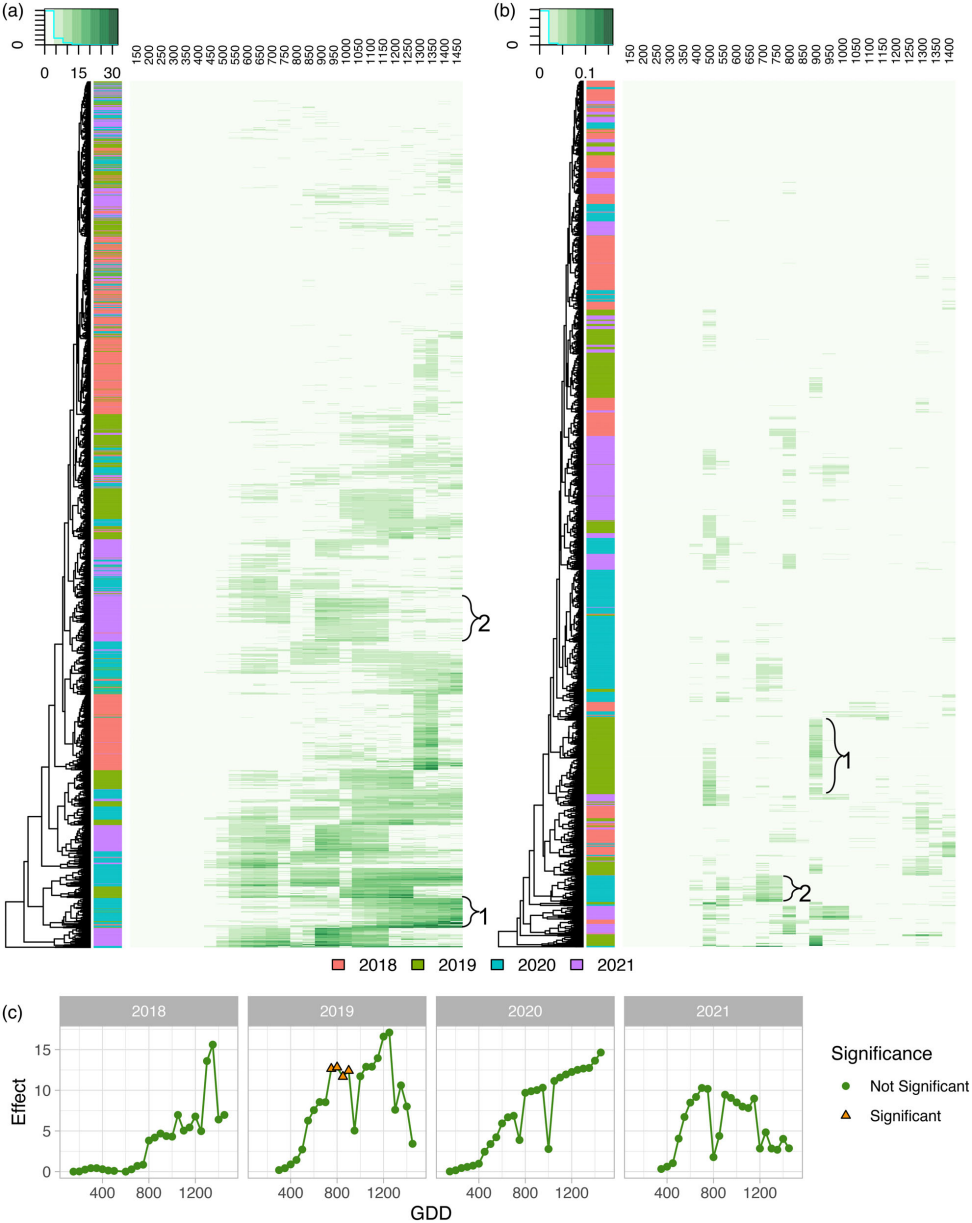
Significant single nucleotide polymorphism (SNP) effects from genome‐wide association study (GWAS) conducted on plant height or growth rate across the growing season. (a) Absolute value of effect of significant SNPs from at least one timepoint‐by‐year assessed across all plant height GWAS iterations in every timepoint throughout the growing season in all four growing seasons. (b) Absolute value of effect of significant SNPs from at least one timepoint‐by‐year assessed across all growth rate GWAS iterations in every timepoint throughout the growing season in all four growing seasons. (c) Absolute value of effect of an example significant SNP from plant height located on Chr2 at position 169 140 523 in the B73 v4 reference genome assembly throughout each of the growing seasons.

The lack of overlap in significant SNPs over time and years is likely because the threshold for significance in GWAS studies is high (Fadista et al., 2016), and the effects of individual loci for our highly quantitative traits were relatively low (Figure 4a,b). Still, we were curious if patterns of effect sizes could be observed over time regardless of the significance of the marker at each timepoint as that may indicate importance at more timepoints regardless of statistical significance. For this analysis, all SNPs that were observed to be significant in at least one timepoint‐by‐year combination for either height (Figure 5a) or rate (Figure 5b) were assessed across all timepoints and years. A group of significant SNPs for plant height had gradually increasing effect size over the growing season (Figure 5a—Bracket 1 and Figure 5c), which may indicate these SNPs are important throughout growth, and contribute to overall terminal height. This is similar to a pattern observed for an SNP found significant with temporal plant height in Adak, Anderson, and Murray (2023). Some of these SNPs had the same effect size patterns over multiple years. Other SNPs had a larger effect size during mid‐vegetative growth without any large increases across the timepoint (Figure 5a—Bracket 2). As would be expected due to the few SNPs significant over multiple years, there are also a lot of SNP‐year combinations that do not appear to have much of an effect at any point in the growing season. This variation in effect patterns is consistent with highly variable patterns across time found in previous work using a population of recombinant inbred lines (Adak, Anderson, & Murray, 2023).

The effect sizes of SNPs significantly associated with growth rate throughout the growing season had very different patterns from what was observed for height (Figure 5a,b). Growth rates clustered more by year and the effects were observed for very short periods of time without any indication of increasing or decreasing effect size throughout development. For instance, a group of SNP‐year combinations from 2019 shows their largest effect size at the 900–950 growth rate (Figure 5b—Bracket 1). This coincides with a lodging event that took place that year (Tirado et al., 2021). Another group of SNP‐year combinations mostly from 2018 to 2020 had their largest effect from 700 to 800 GDDs, which coincided with the early to mid‐exponential growth phase in those years (Figure 5b—Bracket 2). These results are consistent with the ANOVA results in which a larger percentage of the observed variation was explained by year for growth rate (Figure 1c). The high variability of effect size linked to growth rate and the short time frames in which SNPs are significant highlight the extreme complexity of this trait and the major impact of immediate environmental conditions.

### Plant height is more genetically controlled, while canopy cover is more influenced by the environment

Canopy cover, or the percentage of ground covered by overhanging aboveground plant material, is another important trait contributing to yield potential, biomass accumulation, light interception, and weed suppression (Campillo et al., 2008; Jannink et al., 2001; Purcell, 2000; Xavier et al., 2017). In order to maximize the competitive benefits of canopy cover, most selections push toward higher cover percentages early in the growing season. Historical methods of measuring canopy cover are often time‐consuming and difficult to complete (Campillo et al., 2008; Purcell, 2000). UAV collection of canopy cover increases both the speed and precision of collection by reducing the amount of time spent in the field and the objectivity of the observer and sun‐angle (Campillo et al., 2008; Purcell, 2000). Similar to plant height, canopy cover increases throughout the vegetative growth stage, making it another potential trait for which growth patterns over developmental time can be assessed for genetic and environmental contributions.

To facilitate a direct comparison of the growth patterns between plant height and canopy cover, comparable growth curves were generated for canopy cover data extracted from the same flight data that plant height was extracted (Figure S7) (Cooper et al., 2024), and values in 50 GDD windows throughout development were predicted from the curves (Table S13). Grouping the canopy cover growth curves using fuzzy c‐means clustering revealed different types of patterns than were observed for plant height, with relative ranks among plots remaining more consistent throughout development (Figure S8). Differences in the Fréchet distances between the same genotypes over multiple years were also observed (Figure 3; Figure S9), with larger average distances for canopy cover (average 0.575 for canopy cover versus 0.415 for plant height). This indicated that canopy cover is more influenced by interactions with environmental factors than plant height. Indeed, when variance was partitioned, the genotype‐by‐year interaction consistently explained 12–20% of the variation; while in plant height, genotype‐by‐year explained less than 10% of the variation for most of the growing season (Figure 1b; Figure S10). During the early exponential growth phase, year by itself explained the largest percentage of the variation in canopy cover while heritability was at its lowest indicating that environment regardless of genotype has a large impact on canopy coverage variation. Similarly to the Fréchet distance, the variation due to year suggested that canopy coverage is a good indication of the variation in environmental conditions present within the growing season.

### Plant height and canopy cover are not predictive of one another and contribute complementary information throughout the growing season

**Figure 6:**
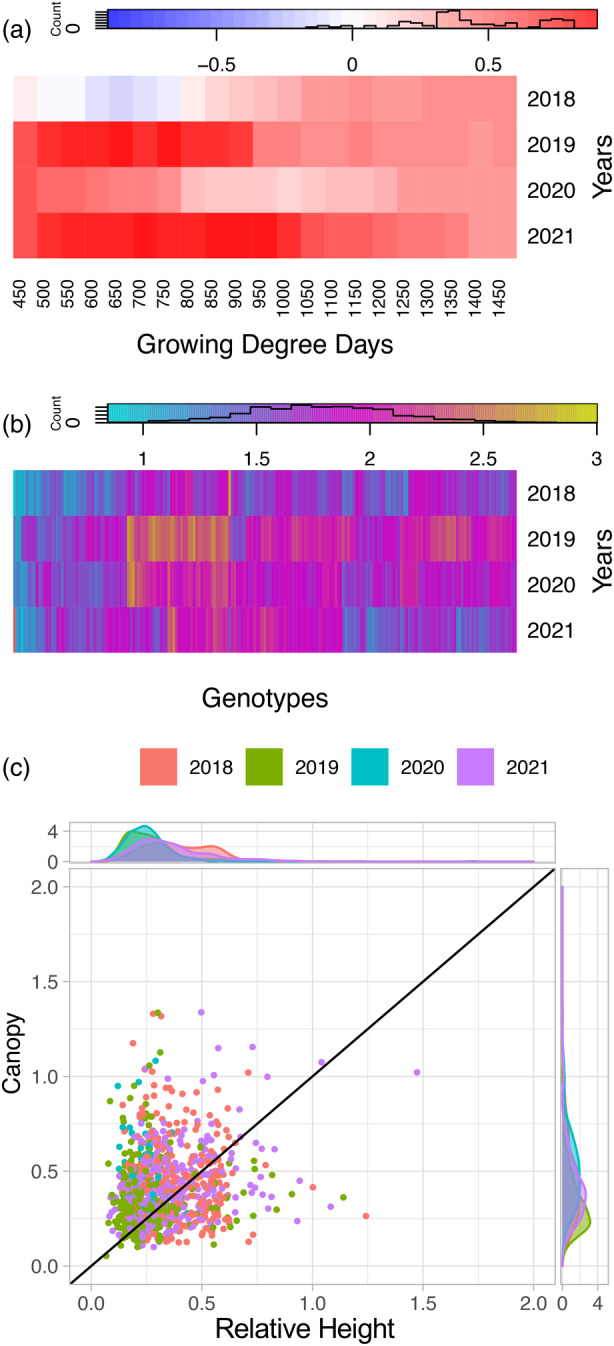
Relationships between plant height and canopy cover. (a) Pearson correlation between plant height and canopy cover across all plots at each growing degree day (GDD) interval for each year. (b) Fréchet distance between the plant height growth curve and canopy cover growth curve within the same plot. (c) Fréchet distance between experimental entry replicates for plant height versus Fréchet distance between experimental entry replicates for canopy cover.

As canopy cover is more influenced by year and genotype‐by‐year interactions, it would be expected to observe differences in plant height and canopy cover progression throughout the season. To test this, direct comparisons of plant height and canopy cover were done. The correlation between plant height and canopy cover within a plot at each GDD interval was highly variable from year to year with a high correlation in early 2019 and 2021 and a low to negative correlation in early 2018 and mid 2020 (Figure 6a). These differences were likely due to environmental variation that differentially affected plant height and canopy cover with the differences between plant height and canopy cover being genotype dependent and canopy cover overall being more variable. To further assess these differences, Fréchet distances were calculated between plant height and canopy cover within each plot (Figure 6b). While many genotype distances (*n* = 169) were consistent across years with a standard deviation less than 0.2, some genotypes showed higher values in some years compared to others, with 2019 having the largest distances. This result was unexpected, as 2019 had a high to average correlation between plant height and canopy cover at individual points in time, but showed the largest individual Fréchet distances, which assess differences in the overall growth curve. This remained true when including all within‐plot comparisons regardless of presence in all 4 years. It is possible the Fréchet distance between plant height and canopy cover identified genotypes more affected by the lodging event that occurred during that year or were more influenced by genotype‐by‐year interactions.

To further assess the difference in environmental responsiveness to within‐year spatial variation, we calculated Fréchet distances between replicates within a year for both plant height and canopy cover. Consistent with the between‐year observations for the same genotype in the same year, replicates had more similar growth curves for plant height than for canopy cover (Figure 6c). This supports that depending on the trait in which growth curves are made, different aspects of plant stability/plasticity will be captured and that in this case canopy cover may be more beneficial in assessing plant responsiveness to environmental variation.

## CONCLUSIONS

Understanding both the genetic basis and phenotypic plasticity of complex traits such as plant height will only increase in importance as our climate becomes increasingly unpredictable. The advent of high‐throughput phenotyping has and will continue to make the necessary data collection for such a task possible. In this study, we provided an in‐depth analysis of the genetic and environmental factors that influenced plant height and growth rate in maize, as well as the methodology for understanding the genetic basis of temporal trait variation. The use of temporal traits in combination with metrics of stability, such as Fréchet distances, gave more information about genotypic plasticity than terminal traits alone and was beneficial to understanding how plants respond to different environments. The genetic architecture of plant height was further underscored by numerous significant SNPs associated with plant height and growth rate throughout the growing season and illustrated the need for a better understanding of temporal traits and implementation for crop improvement.

Overall this study enhanced our understanding of the genetic basis of trait variation in maize by demonstrating the significant interplay between genetics and environment. The findings emphasize the importance of considering temporal dynamics in plant growth studies, which could inform breeding programs aimed at improving crop resilience and performance under varying environmental conditions.

## MATERIALS AND METHODS

### Experimental field design

A set of 501 diverse inbred lines from the Wisconsin Diversity Panel (Burns et al., 2021; Hansey et al., 2011; Renk et al., 2021) were grown in the summers of 2018, 2019, 2020, and 2021. These trials were planted on May 14, 2018; May 30, 2019; May 7, 2020; and May 6, 2021, at the Minnesota Agricultural Experiment Station in St. Paul, MN. The lines were grown in single‐row plots that were ~6 m long center‐to‐center, with ~1.25 m alleys, ~75 cm row‐spacing, and were planted at a density of approximately 70 000 plants per hectare. All experiments were planted as a randomized complete‐block design with two replicates. Within replicates, genotypes were blocked by flowering time with the earlier lines flowering at approximately 71–80 days after planting and the later lines flowering at approximately 80–87 days after planting. Entries were randomized within the block and replicated. These flowering time blocks within replicates were included to account for variation due to flowering time that often contributes to variation in plant height. The inbred lines B73 and PH207 were planted as checks within each block, with five entries of each check per block.

### Manual plant height data collection

**Figure 7:**
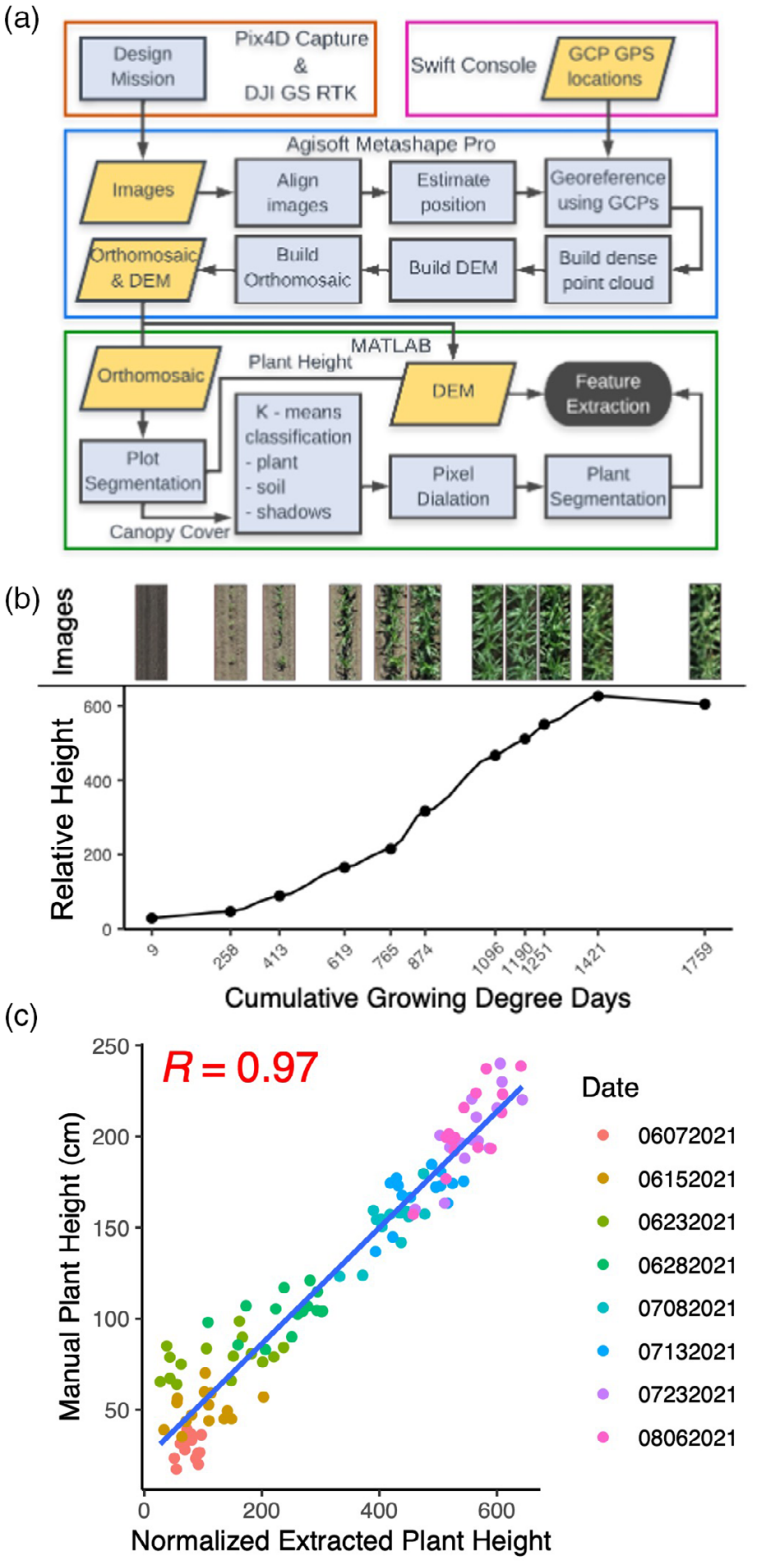
Unoccupied aerial vehicle (UAV) data processing and plant height extraction. (a) Pipeline for image acquisition and feature extraction from UAV images. (b) Extracted plant height normalized across timepoints for a single example plot with segmented views of the plot at each timepoint. (c) Pearson correlation between normalized mean extracted plot plant height and mean manual plot plant height measurements across all dates within a year for height extraction validation.

Manual plant height measurements were collected on the same day as drone flights for experimental plots evaluated in this study in 2020 and 2021 (Table S14), and on other experimental plots not included in this study, but in the same field in 2018 and 2019. Manual plant height measurements were collected on 25 randomly distributed plots in 2020 and 15 randomly distributed plots in 2021. Manual plant heights from 144 plots in 2018 and 240 plots in 2019 were collected as previously reported (Tirado et al., 2021). Across all 4 years, within a plot, manual measurements were collected on five representative plants from the center of the plot using a measuring stick. Plant height was measured as the distance between the ground and the uppermost freestanding vegetative part of the plant until reproductive maturity when height was measured to the top of the tassel. These manual measurements were used to determine the relative accuracy of the extracted heights by correlating the manual measurements to the extracted measurements from the same plots (Figure 7). Within flight, correlations ranged from −0.12 to 0.98 (Figure S11).

### UAV data collection and processing

The experiment was imaged approximately weekly from planting until plants reached terminal height using a DJI Phantom 4 Advanced drone in 2018 and 2019 and a DJI Phantom 4 RTK drone in 2020 and 2021. Images were collected at an altitude of 30 m above ground to achieve a ground sampling distance of approximately 0.82 cm with 80% front overlap and 80% side overlap to maximize reconstruction efficiency. Flights were collected at 14 timepoints in 2018, 27 timepoints in 2019, 12 timepoints in 2020, and 11 timepoints in 2021 (Table S1). Ground targets of known height and half a meter wide were placed around the border of the field for use as ground control points (GCPs). There were 9 GCPs included in 2018, 12 in 2019, 8 in 2020, and 7 in 2021. The real‐world coordinates of these GCPs were collected using real‐time kinematic positioning with a Swift Console (v2.3.17) base station and rover (GNSS compass configuration; Swift Navigation, Inc., 2018) (Table S15).

### Plant height data extraction from aerial images

Images from each flight were processed as previously described (Tirado et al., 2020). Briefly, Agisoft Software (Agisoft Metashape Professional v1.7.5) was used to process the images and generate crop surface models and RGB orthomosaics for each flight. QGIS software (QGIS v3.16, 2021) was used for plot boundary extraction by overlaying a grid based on plot size and spacing and exporting plot coordinates. Custom MATLAB scripts for plant height were used to extract height estimates for individual plots using a previously described exposed alley subtraction method (Tirado et al., 2020) (Figure 7a; Table S2). All extracted plant heights were normalized across flights by comparing the extracted GCP heights (Figure S1) across each flight. This resulted in plant height values that were normalized across time and were, therefore, relative to each other, but not representative of any real‐world units (Figure S1a).

The normalized plant heights were subjected to quality control analysis at the field, plot, and individual data point levels. At the field level, visual assessment of heights across the field in heat maps removed four erroneous flight dates in 2019 and an entire second location in 2020 that were not included in the flight counts above. Plots with less than 10 plants were removed from the analysis due to the decrease in extracted plant height accuracy and the competition from neighboring plots affecting the actual plant height. This filtering step removed 325 out of the original 4160 plots across the four environments. Individual data points (i.e., single plots within a single flight date) were removed if there was a dip in height when the plot height was less than 80% of the previous day and also remained less than the next day, or if the individual data points were classified as a peak when the plot height was more than 120% of the next day while still remaining more than the previous day (*n* = 2273 data points removed as dips or peaks). Entire plots were removed from a location if the plot height dipped or peaked on three or more occasions (*n* = 172 plots removed). In total, 8238 individual data points (1201 in 2018, 3743 in 2019, 2029 in 2020, and 1265 in 2021) were removed. After these filtering steps, 3663 of the original 4160 plots across the four years had at least nine individual measurements within the year and were retained for downstream analysis (Table S3).

### Weather data and growing degree day unit calculations

Daily minimum and maximum temperature and precipitation data were collected from the University of Minnesota St. Paul weather station (Station ID: 218450) (Minnesota Department of Natural Resources, 2024). GDDs were calculated for each date utilizing the equation: $\mathrm{GDD}=\left[\left(T_{\max}+T_{\min}\right)/2\right]-50$, where GDD is the growing degree day units accumulated for a single day and *T*_max_ and *T*_min_ are the maximum and minimum recorded air temperature values in degrees Fahrenheit for the given date. Temperature values above 30°C were adjusted to 30°C and values below 10°C were adjusted to 10°C since corn growth rates do not increase or decrease outside of this range. The cumulative sum of GDDs for data collection dates was calculated as the sum of GDDs for all days between the planting date and the flight date (Figure S1b). Daily data for 18 weather features were obtained for each year using envirotypeR in R v.3.6.2 (R Core Team, 2019) from planting to approximate flowering time at 90 days after planting (Table S5). Correlations between years were calculated by converting the environmental data into pairwise Euclidean distance matrices using the “daisy” function in the “cluster” package v.2.1.4 (Maechler et al., 2023) and then using the “mantel.rtest” function in the “ade4” package v.1.7‐22 (Dray & Dufour, 2007) in R v.3.6.2 (R Core Team, 2019) to calculate the correlations between each pair of years.

### LOESS regression modeling

Separate LOESS regression models were fit to the RGB extracted plant heights for each plot using all flight timepoints that passed the above filtering criterion using the “loess” function in the “stats” package v.3.6.2 in R v.3.6.2 (R Core Team, 2019). A span of 0.15 to 0.35 was used for regression modeling based on an error matrix developed with 100 folds to determine the best‐fitting span for each year (Figure S12). Plant height values for every 50 GDDs of the growing season for each model (plot within a year) were predicted using the fitted model and trimmed to 150 to 1450 GDDs. These values were used to calculate the rate of growth between two‐timepoints as the slope between the two points (Table S4).

### Analysis of variance

The variation in extracted plot plant height values and growth rates was partitioned into genotype, year, replicate nested within year, block nested within replicate within year, and genotype by year interaction with all terms treated as random effects with the linear model plant height ~ *g* + *y* + *y*/*r* + *y*/*r*/*b* + *g*:*y* + *ε*, where *g* is genotype, *y* is year, *r* is replicate, *b* is block, and *ε* is residual. To test the significance of the sources of variation of the linear model on plant height and growth rate, the “Anova” function from the “car” package v.3.1‐1 (Fox & Weisberg, 2019) in R v.3.6.2 (R Core Team, 2019) was used. The “tidy” function in the “broom” package v.1.0.3 (Robinson et al., 2024) was used to capture the output of the anova and calculate the percent variance explained by each source of variation in R v.3.6.2 (R Core Team, 2019). Narrow sense heritability (*h* ^2^) was calculated on an entry‐mean basis using the following equation:

$$h^{2}=\sigma_{g}^{2}/(\sigma_{g}^{2}+\frac{\sigma_{g\times e}^{2}}{e}+\frac{\sigma_{\varepsilon }^{2}}{\mathrm{re}})$$

where $\sigma_{g}^{2}$ is the genotypic variance, $\sigma_{g\times e}^{2}/e$ is the genotype × environment interaction variance, $\sigma_{\varepsilon }^{2}$ is the error variance, e is the number of environments, and r is the number of replications. Variance for this equation was calculated using the “VarCorr” function from the “nlme” package v. 3.1–160 (Pinheiro et al., 2024) in R v.3.6.2 (R Core Team, 2019).

### Clustering growth curves

The LOESS curves were soft clustered within‐year using fuzzy c‐means clustering using the “ppclust” package v.1.1.0.1 (Cebeci, 2019) in R v.3.6.2 (R Core Team, 2019) (Table S6). The curves were grouped into individual clusters based on the goodness of fit to each cluster, with each curve placed in the group with the highest percentage fit. Comparisons of the clustering between years were completed to determine the consistency of growth curve clustering using an upset plot showing the count of genotypes shared in each cluster type using the “upset” function in the “UpSetR” package v.1.4.0 (Gehlenborg, 2019). A Fréchet distance was calculated between replicates of the same genotype within each year and across years using the “TSdist” package v.3.7.1 (Mori et al., 2016) in R v.3.6.2 (R Core Team, 2019) with replicates within a year averaged at each timepoint to compare across years (Table S8). The genotypes were divided into groups based on the average Fréchet distance and terminal height variance within this dataset. Anything in the lowest 20 percent (*n* = 100) was considered to be low (Fréchet distance less than 0.330 or plant height variance less than 1150). Anything in the highest 20 percent was considered high (Fréchet distance greater than 0.510 or plant height variance greater than 4075).

### Genome‐wide association studies

Genome‐wide association studies were completed as previously described (Burns et al., 2021; Renk et al., 2021). In brief, GAPIT v.3 (Wang & Zhang, 2021) was used to transform genomic data into numeric format, generate a genotypic map dataset, and a PCA covariates dataset. SNP data was obtained from a previous study (Qiu et al., 2021) and filtered as previously described for minor allele frequency, missing data, and LD within 10 kb (Renk et al., 2021). A separate GWAS was performed for each of the predicted GDD timepoints, derived growth rates, and normalized extracted terminal heights in each year using BLUPs extracted from the random effects model described above within each year. The genetic map, filtered numeric genomic dataset, and filtered BLUP datasets were used to permute a suggested *P*‐value for each GWAS using the “FarmCPU.P.Threshold” function in FarmCPU v.1.02 (Liu et al., 2016) in R v.4.0.4 (R Core Team, 2019) as previously described (Renk et al., 2021). SNPs in linkage disequilibrium with each other were identified using PLINK v.1.90b6.10 (Chang et al., 2015; Purcell et al., 2007; Shaun Purcell, 2024). SNP effect over time was plotted in a heatmap using “heatmap.2” from the “gplots” package v.3.1.3 (Warnes et al., 2024) (R Core Team, 2019), and the SNP effects were clustered using the default “hclust” function from heatmap.2 (Table S9). The nearest gene to a SNP was identified relative to the Zm‐B73‐REFERENCE‐GRAMENE‐4.0 Zm000014 Gene Set from MaizeGDB (Maize B73 RefGen_v4) (Monaco et al., 2013) using the closest function in BEDTools v2.29.2 (Quinlan & Hall, 2010) with default parameters. Functional annotations for this gene set were downloaded from Gramene (ftp://ftp.gramene.org/pub/gramene/CURRENT_RELEASE/gff3/zea_mays/gene_function/B73v4.gene_function.txt) on 8‐Nov‐2018. Overlap with significant regions from previous studies (Adak, Murray, Anderson, et al., 2021; Mazaheri et al., 2019; Wang et al., 2019; Zhang et al., 2019) was completed using the “window” function in bedtools version 2.31.1 (Quinlan & Hall, 2010) with SNPs within 100 kb considered overlapping. Enrichment of gene ontology terms was assessed using the agriGO online application (Tian et al., 2017).

### Genomic prediction

Genomic prediction was completed at each 50 GDD timepoint from 150 to 1450 GDDs using the filtered numeric genomic dataset described above. Best linear unbiased estimates (BLUE) were calculated for each genotype within a timepoint across all years with all terms treated as random effects except for genotype, which was a fixed effect, using the “lmer” function from “lme4” package v.1.1‐32 (Bates et al., 2015) with the linear model: plant height ~ *g* + *y* + *y*/*r* + *y*/*r*/*b* + *g*:*y* + *ε*, where *g* is genotype, *y* is year, *r* is replicate, *b* is block, and *ε* is residual. The fixed effects of this model were extracted for the BLUEs using the “fixef” function from the “nlme” package v.3.1‐160 (Pinheiro et al., 2024). BLUEs were then separated into training and testing sets with 80% of the genotypes in the training set and 20% in the testing set 25 separate times for each timepoint. Genomic prediction was completed using the “mixed.solve” function from the “rrBLUP” package v.4.6.2 (Endelman, 2011). Accuracy of the genomic prediction method was ascertained using the “cor” function from the “stats” package v.4.2.2 in R v.4.2.2 (R Core Team, 2019).

### Canopy cover data analysis

The same flights that were processed for plant height in this study were previously processed for canopy cover using the same crop surface models, RGB orthomosaics, and plot boundaries described above (Cooper et al., 2024). LOESS regression models were fit using the same methods described above for plant height and point and growth rate values in 50 GDD windows were predicted from the models (Table S13).

As with plant height, a Fréchet distance was calculated between replicates of the same genotype within years and between the same genotype across years for canopy cover using the “TSdist” package v.3.7.1 (Mori et al., 2016) in R v.3.6.2 (R Core Team, 2019) (Table S8). Soft clustering was completed within‐year using fuzzy c‐means clustering using the “ppclust” package v.1.1.0.1 (Cebeci, 2019) in R v.3.6.2 (R Core Team, 2019) (Table S6). Similarities in clustering were evaluated in an upset plot using the “UpSetR” package v1.4.0 (Gehlenborg, 2019) in R v.3.6.2 (R Core Team, 2019). *z*‐Scores were calculated for both plant height and canopy cover using the following equation: $z=\left(x-\mu \right)/\mathrm{SD}$, where *z* is the *z*‐score for the individual plot, *x* is the individual plot plant height or canopy cover value, *μ* is the average value across all retained data points for plant height or canopy cover, respectively, and SD is the standard deviation across all retained data points for plant height or canopy cover, respectively, in order to make plant height and canopy cover comparable in scale to one another. Pearson correlations and Fréchet distances between plant height and canopy cover in the same experimental plot were completed using the “cor” function in the “stats” package v.3.6.2 and the “TSdist” package v.3.7.1 (Mori et al., 2016) in R.v.3.6.2 (R Core Team, 2019).

## CONFLICT OF INTEREST

The authors have no relevant financial or non‐financial interests to disclose.

## DATA AND CODE AVAILABILITY STATEMENT

Scripts and files used to generate and analyze data are available on GitHub at https://github.com/HirschLabUMN/WiDiv_Drone_Height.git. All data including the UAV‐derived plot height values, UAV‐derived canopy cover values, orthomosaics, DEMs, plot boundary files, and mask files for each date of UAV data collection, cumulative GDDs calculated for each date of data collection, manual height data, and weather data has been made available at the Digital Repository for U of M (DRUM) at https://doi.org/10.13020/SKJN‐QX31.

## Supporting information


**Figure S1.** Normalization of extracted plant height values. (a) Example values extracted from GCP bounding boxes to identify height values for the ground and tops of the GCPs (06022019). (b) Accumulation of growing degree days (GDDs) throughout the growing season for each year. Extracted plant height values across the growing season for all plots with (c) raw data (before any cleaning), (d) normalized data (before cleaning but normalized across dates using GCP heights), and (e) clean data (normalized data with erroneous plots removed before analysis). Dates on the *x*‐axis are in the form MMDDYYYY.
**Figure S2.** Accuracy of genomic prediction throughout the growth season. Heritability was calculated on an entry‐mean basis. RMSE increased throughout development due to the increased scale of plant height throughout development.
**Figure S3.** Plant height growth curves and precipitation accumulation throughout the growth season. The LOESS growth curves for all plots within each year on the left *y*‐axis and the accumulation of precipitation throughout the growing season in black on the right *y*‐axis. (a) 2018, (b) 2019, (c) 2020 (d) 2021.
**Figure S4.** Density of largest percentages for determination of number of clusters to use for Fuzzy c‐means clustering. The density of the largest value of goodness of fit for each curve to any single cluster when the curves are broken into 2, 3, 4 or 5 clusters with the value of the largest percentage of fit on the *x*‐axis. These graphs were used to determine how many clusters fit the data best. (a) 2018, (b) 2019, (c) 2020, (d) 2021.
**Figure S5.** Fuzzy c‐means clustering of plant height growth curve values. Fuzzy c‐means clusters of LOESS growth curves with shading equating goodness of fit for each curve into the specified cluster separately for each year.
**Figure S6.** Pearson correlation between environmental similarity and average Fréchet distance. Fréchet distance between years for all genotypes with data in both years were averaged within each pair of years. Environmental correlations were calculated based on daily values for 18 weather parameters from planting to approximate flowering at 90 days after planting.
**Figure S7.** Temporal canopy cover growth curves. LOESS curves of canopy cover broken into three phases of the growing season based on the performance of genotypes in each year.
**Figure S8.** Fuzzy c‐means clustering of canopy cover growth curve values. Fuzzy c‐means clusters of LOESS growth curves with shading equating goodness of fit for each curve into the specified cluster separately for each year.
**Figure S9.** Fréchet distances for each genotype present in all 4 years. Pairwise Fréchet distance values comparing canopy cover growth curves of the same genotype across different years.
**Figure S10.** Temporal canopy cover analysis of variance. Percent variance explained and heritability at each canopy cover timepoint throughout the growing season.
**Figure S11.** Validation of extracted plant height. Pearson correlation of normalized mean extracted plot plant height to mean manual plot plant height measurements. The date of each flight is indicated in the *x*‐axis label in the form MMDDYYYY.
**Figure S12.** Span choices for LOESS curve fitting each year. Mean cross‐validation error for each possible span from 0.15 to 0.95 for each year with the best span for each year red (left plots). Growth rates with loess curves fit using the best span (right plots).

**Table S1.** Year, date of year, growing degree days after planting, and notes for each flight used in this study.

**Table S2.** Extracted raw data for each retained flight date (MMDDYYYY) before normalization or individual plot removal identified by genotype and replicate.

**Table S3.** Cleaned and normalized extracted data for each flight date (MMDDYYYY) before loess curve fitting identified by genotype and replicate.

**Table S4.** Clean, normalized, extracted plant height values predicted from LOESS curves for all retained flights. Plant height represented by ###_GDD and growth rate represented by ###_###_GDD.

**Table S5.** Daily environmental information pulled from envirotypeR for each year from planting to 90 days after planting. T2M – temperature at 2 m; T2M_MAX – maximum temperature at 2 m; T2M_MIN – minimum temperature at 2 m; PRECTOT – precipitation corrected (mm/day); WS2M – wind speed at 2 m; RH2M – relative humidity at 2 m; T2MDEW – dew/frost point at 2 m; *n* – actual duration of sunshine (hours); *N* – daylight hours (hours); RTA – extraterrestrial radiation (MJ/m^2^/day); SRAD – solar radiation (MJ/m^2^/day); SPV – slope of saturation vapor pressure curve (kPa/°C); VPD – vapor pressure deficit (kPa); ETP – potential evapotranspiration (mm/day); PETP – deficit by precipitation (mm/day); GDD – growing degree day (°C/day); FRUE – effect of temperature on radiation use efficiency (from 0 to 1); T2M_RANGE – daily temperature range (°C day).

**Table S6.** Goodness of fit for each genotype into each cluster for plant height and canopy cover across all 4 years.

**Table S7.** Number of genotypes partitioned into each fuzzy c‐means cluster.

**Table S8.** Fréchet distances within genotype for plant height and canopy cover across different years and between plant height and canopy cover within the same year.

**Table S9.** All significant SNPs with p values and effect sizes for every GWAS iteration.

**Table S10.** Functional annotation of the nearest gene to each of the significantly associated loci found across all GWAS timepoints.

**Table S11.** Enriched gene ontology terms for loci that were significantly associated with height or growth rate during the exponential growth phase.

**Table S12.** Metadata on significant SNPs in this study. Number of times significant includes the number of timepoints a SNP was statistically significant in any timepoint‐by‐year combination for both plant height and growth rate. An SNP was considered to overlap a previously identified QTL if it was within 100 kb of the previous SNP or QTL region.

**Table S13.** Extracted percent canopy cover from LOESS curves fit to all retained flights after quality control filtering.

**Table S14.** Manual plant height measurements (cm) for given plots identified by plot number on the given date (MMDDYYYY).

**Table S15.** GPS location of all ground control points (GCPs) across years.
